# Zebra Stripes through the Eyes of Their Predators, Zebras, and Humans

**DOI:** 10.1371/journal.pone.0145679

**Published:** 2016-01-22

**Authors:** Amanda D. Melin, Donald W. Kline, Chihiro Hiramatsu, Tim Caro

**Affiliations:** 1 Department of Anthropology, Washington University in St. Louis, St. Louis, Missouri, 63130, United States of America; 2 Departments of Anthropology & Archaeology and Cell Biology & Anatomy University of Calgary, Calgary, Alberta T2N 1N4, Canada; 3 Departments of Psychology and Surgery (Ophthalmology), University of Calgary, Calgary, Alberta T2N 1N4, Canada; 4 Department of Human Science, Faculty of Design, Kyushu University, Fukuoka, 815–8540, Japan; 5 Department of Wildlife, Fish and Conservation Biology, University of California Davis, Davis, California 95616, United States of America; University of Sussex, UNITED KINGDOM

## Abstract

The century-old idea that stripes make zebras cryptic to large carnivores has never been examined systematically. We evaluated this hypothesis by passing digital images of zebras through species-specific spatial and colour filters to simulate their appearance for the visual systems of zebras’ primary predators and zebras themselves. We also measured stripe widths and luminance contrast to estimate the maximum distances from which lions, spotted hyaenas, and zebras can resolve stripes. We found that beyond ca. 50 m (daylight) and 30 m (twilight) zebra stripes are difficult for the estimated visual systems of large carnivores to resolve, but not humans. On moonless nights, stripes are difficult for all species to resolve beyond ca. 9 m. In open treeless habitats where zebras spend most time, zebras are as clearly identified by the lion visual system as are similar-sized ungulates, suggesting that stripes cannot confer crypsis by disrupting the zebra’s outline. Stripes confer a minor advantage over solid pelage in masking body shape in woodlands, but the effect is stronger for humans than for predators. Zebras appear to be less able than humans to resolve stripes although they are better than their chief predators. In conclusion, compared to the uniform pelage of other sympatric herbivores it appears highly unlikely that stripes are a form of anti-predator camouflage.

## Introduction

The functional significance of the unique high-contrast black and white stripes of zebra species is a subject of considerable debate. Historically, four major hypotheses have been offered to account for the function of zebra stripes: an antipredator defense operating either through crypsis, aposematism, or confusion of predators [1–3]; a means of reinforcing social bonds [4]; a defense against ectoparasites [5, 6]; and as a means of cooling [7]. The predation hypothesis was the first to receive attention with some of the great Victorian biologists debating whether stripes were cryptic or conspicuous in natural settings. For example, Wallace ([8], p. 220) remarked “Mr. Francis Galton, who has studied these animals in their native haunts, assures me, that in twilight they are not at all conspicuous, the stripes of white and black so merging together into a gray tint that it is very difficult to see them at a little distance.”, whereas Darwin ([9], p. 832) wrote “the zebra is conspicuously striped, and stripes on the open plains of South Africa cannot afford any protection”. The idea that zebras, highly conspicuous to us when relatively near, are somehow cryptic at a distance is perhaps the most counterintuitive yet long lasting idea about zebras’ stripes. For instance, Godfrey and colleagues wrote in 1987: “Zebras have always presented a problem to those interested in cryptic colouration, because in most types of open country in daylight they are exceedingly easy to see despite any disrupting effect of the stripes on their body outline. It is only in the gathering twilight when the resolving power of the eye decreases and the stripes merge into a uniform grey, that zebras become cryptic” [10].

Unfortunately, we still have little idea of how predators or even conspecific zebras view striped coats in natural situations. This problem is exacerbated by humans’ anomalously acute photopic spatial vision that far surpasses the ability of non-primate mammals to resolve detail, an ability that may have led us to overestimate the conspicuity of striped pelage for other species [11]. In this, lighting condition is a key issue because the bright-light sensitive cone photoreceptors of the retina provide a far more detailed image of a scene than do the low-light rods. Such functional dualism of the retina means that spatial resolution is far better in bright daylight conditions than as night approaches. Yet, predators are crepuscular, and far more likely to attack at dusk or nighttime [12, 13]. Here we begin to address this gap in our understanding of the adaptive significance of zebra stripes by using a perceptual approach based on the spatial and colour vision of the potential receivers. To test the premise of the crypsis hypothesis, we estimate, for the first time, the ability of lions (*Panthera leo*) and spotted hyaenas (*Crocuta crocuta*) (the chief predators of zebras), and of zebras themselves (*Equus* sp.) to resolve zebra stripes, relative to the ability of humans. To do so, we used spatial-vision-based digital image filtering techniques to compare images of zebras and other prey animals as they might be seen in high-, intermediate- or low-light conditions.

### Spatial Vision and Digital Image-Processing

Spatial vision refers generally to the ability to resolve spatially defined features. The term commonly applies to the perception of stationary two-dimensional luminance patterns measured by resolution acuity or the contrast sensitivity function (CSF). By testing the level of contrast needed to resolve harmonic targets—typically sinusoidal gratings that vary in spatial frequency—the CSF provides a more comprehensive measure of spatial vision than does acuity, a measure of the finest high contrast detail (i.e., highest spatial frequency) that can be resolved. Consequently, the CSF is more useful for predicting performance on real-world tasks [14–16]. In the context of a linear systems application to spatial vision, CSF testing allows for the analysis, transformation and perceptual representation of complex digital scenes.

As noted by Shapley [17], linear and nonlinear systems analyses are very useful tools for understanding the visual system. Ginsburg [18] described three general applications of linear systems analysis in visual research: (1) a quantitative description of the spatial properties of a display, (2) a general model of how the visual system processes spatial information, and (3) a basis for filtering spatial information selectively. In the present study, a Fourier convolution procedure was carried out in which natural world scenes were modified by digital weighting functions (i.e., the CSFs) representative of the spatial vision abilities of a lion, spotted hyaena, zebra or human in high, intermediate or low light conditions. The resulting digital images were then passed through a dichromatic colour vision filter to represent the net luminance and chromatic contrast likely to be experienced by the non-humans considered in this study and a trichromatic filter to represent standard human vision.

### Goals of Study

The possible anti-predator benefits of zebra striping founded on the visual systems of different observer species have not been evaluated (but see [10]). Here, we estimate the ability of lions and spotted hyaenas (the two chief predators of zebras), and of zebras themselves to resolve zebra stripes, relative to humans by (1) calculating distance limits for the resolution of zebra stripes by the different species, and (2) applying spatial and colour vision filters to digital images of zebras and other prey animals at different distances to simulate their appearance under higher-, intermediate- and lower-light conditions. The luminance levels were selected to estimate relative differences between vision under (a) bright, daylight conditions (photopic), (b) dim, dawn/dusk conditions (mesopic) and (c) dark, night conditions (scotopic). However, recognizing that the reader will most likely view these images photopically, the image light levels were kept within the photopic range. Because we use static digital images, we address the conspicuity of stationary zebras.

If zebra stripes can be resolved by predators at a distance or under low lighting conditions or both, this would support a critical component of the argument that striping is a form of camouflage, albeit a rather unique one [19]. Striping might mediate its effect through background matching against woodland habitat [20] or disruptive colouration by breaking up the body’s outline [1]. If, however, stripes were difficult for predators to resolve when far away and/or under poor light, this would suggest that zebra striping does not act in a camouflage capacity, although this does not rule out a role of stripes in signalling the dangers of attacking–bites, sharp kicks–from short distances. If stripes are more conspicuous to zebras than their predators, it might implicate their importance in intraspecific interactions.

## Materials and Methods

### Spatial and Luminance Characteristics of Zebra Stripes

Zebra stripes, although of irregular width, can broadly be thought of as naturally occurring square wave gratings, the frequency (cycles per degree visual angle; cpd) of which varies according to body region and viewing distance. A species’ acuity can be measured using gratings of high contrast, typically 0.5–0.8, of varying spatial frequency [21–23]. Using acuity values based on behavioural studies or projections based on reconstructed CSFs, we calculated the maximum distance at which the thinnest and widest stripes on the body of adult plains (*Equus burchellii*), mountain (*E*. *quagga*) and Grevy’s zebras (*E*. *grevyii*) could be resolved by different predators, other zebras, and humans. We also measured the luminance of the black and white stripes of live captive zebras to quantify the mean Michelson luminance contrast [(L_max_—L_min_)/(L_max_+L_min_)]_,_ where L = luminance, in ambient conditions ranging from daylight to twilight.

Luminance of the stripes of live zebras (*E*. *grevyii*) and the immediate surrounding area (sky, grass, dirt) above, below, to the left and to the right of the animal was recorded by ADM using an LS-110 Minolta Spot Photometer with a one-degree acceptance angle on August 29^th^ 2012 at the Calgary Zoo. Luminance under photopic conditions was recorded at 2:50 pm under full sun. Luminance under photopic (sun with light cloud) then mesopic (civil twilight) conditions was measured from 7:50 pm past sunset (8:27 pm) until ambient levels fell below the range of the meter at 8:43 pm (civic twilight end at 9:01 pm). Zebras were measured in both open (full sun) and shaded (enclosure structures, trees) conditions. In the former case, the stripe luminance was measured above and below the natural body shadow. Absolute luminance was higher in stripes exposed to full sun but luminance contrast was highly conserved across conditions (S1 Dataset). Data collection was approved by the Calgary Zoo’s Biological Research Review Committee.

Stripe width measurements were taken from a total of 9 preserved study skins from the Los Angeles County Museum of Natural History (2 *Equus burchellii*, 1 *E*. *zebra*, 1 *E*. *grevyii*), California Academy of Sciences (1 *E*. *burchellii*, 2 *E*. *grevyii*), and UC Berkeley’s Museum of Vertebrate Zoology (2 *E*. *burchellii*) (H. Walker, S2 Dataset). To obtain stripe widths, the body was divided into 7 sections: forehead (right to left tear duct), cheek (lip to ear), neck (ear to forelimb), flank (end of forelimb to start of hindlimb), forelimb (hoof to site where limb leaves body), and hindlimb (hoof to site where limb leaves body), and rump (hindlimb to anus). A metre stick (mm) was centered at the edge of each section and aligned to pass the section’s stripes perpendicularly and widths of stripes were measured. For plains and mountain zebra pelts, the rump measurement was taken by running the metre stick from the start of the hindlimb to initial site of curvature on the back. For Grevy’s zebra pelts, the measurement was divided into two parts to account for directional changing of the stripes. The metre stick was first run from hindlimb start to “Y” shape, then from “Y” to anus. Permission to handle zebra pelts was granted by the collaborating museums.

### Collection of Digital Photographs

Photographs of live adult and subadult plains zebras, waterbuck (*Kobus ellipsiprymnus*), topi (*Connochaetes taurinus*) and impala (*Aepyceros melanopus*); pelts of plains zebras, wildebeest (*Alcelaphus buselaphus*) and impala; and of two-dimensional life-size models of striped and “solid” zebras constructed of plywood and painted with glossy paint, were taken in the field in Katavi National Park, western Tanzania in August and September 2012 by TC. Following guidelines for using digital photography to study animal colouration [24], photographs were taken with a 300-mm zoom telephoto lens mounted on a Nikon D50 camera set at 70 mm in manual mode with EV default steps set at 1/3^rd^, on auto focus, and shot in RAW format. Objects (animals, pelts or models) were photographed from 100–200 m away; precise distances were subsequently recorded using a rangefinder. Within 30 s after a photo was taken, TC went to the object’s location and erected a standard 24-square GretagMacbeth Colour Chart 1 m above ground. He then returned to the vehicle and photographed the colour checker card with the same zoom (70 mm) aperture and shutter speed settings. We used the PictoColor in Camera Plug-In for Photoshop to achieve consistent colour profiles across images. To overcome digital photography dynamic range limitations under dark conditions, the photographs were taken over a range from of light levels from high mesopic to high photopic. A complete dataset of all original and filtered images is available at the Harvard Dataverse, DOI: 10.7910/DVN/NZNAEH). Field research permission was granted by the Tanzania Wildlife Research Institute.

### Projecting Maximum Distance of Visual Resolution of Stripes

We calculate the maximum distance at which different species should be able to resolve the widest and narrowest striping found on different body regions of the three zebra species. Given that targets are small when viewed from a distance, we use the formula V = arctan (S/D), where V is the minimum resolvable visual angle in degrees (acuity), S is the width of a stripe, and D is distance. In this study, species-specific acuity measures are extrapolated from similar species for which spatial vision has been measured behaviourally.

#### Simulating spatial, colour and luminance vision: Linear analysis of digital photographs

CSFs have been determined behaviourally under varying illumination conditions for humans and for other species, including carnivores (domestic cats, *Felis catus*) and equids (horses, *Equus caballus*) [11, 23, 25, 26]. Importantly, the inverted-U shape of the CSF of non-primate mammals is highly conserved. CH and ADM estimated the CSFs of lions, spotted hyaenas and zebras by matching a parabolic function to the existing domestic cat data under different lighting conditions, and then extrapolating to our species of interest by shifting the domestic cat CSFs to account for relative differences in eye size and estimated visual acuity (S1 Fig). This procedure is based on the following: (1) animal CSFs in log scale are well characterized by a parabolic function [11, 27]; (2) visual acuity increases with axial eye length [28–30]; (3) the shape of mammalian CSFs is similar for animals without a fovea (i.e. non-anthropoid mammals) [31]; and (4) reliable CSFs of an afoveat mammal under a range of conditions ranging from scotopic through photopic are well described for domestic cats [23].

Our specific approach for predators was as follows: we fitted cat CSFs recorded under the photopic (16 cd/m^2^), mesopic (0.16 cd/m^2^) and scotopic (0.000016 cd/m^2^) conditions [23] to a parabolic function in log scale, with the following coefficients: “a” slope, “b” peak spatial frequency, and “c” peak sensitivity of the function. Visual acuity of the cat was set to 5.81 cpd. This acuity value is broadly consistent with estimates for domestic cats measured in other studies [21, 32–35]. Since the upper limits of acuity for lions and spotted hyaenas are unknown behaviourally, we necessarily estimated the visual acuity (VA) from axial eye diameter (AD; Table 1) using the regression coefficients reported in [30]. Then, we estimated the CSFs of lions, and hyaenas by shifting the b value of the cat CSF by the difference in visual acuity between each species and the cat. For example, b_lion_ = b_cat_ + (VA_lion_—VA_cat_). The slope, which differed across luminance conditions, was held constant among species. This approach is a reasonable first-order approximation as lions and hyaenas have broadly similar retinal morphologies to cats, including topography and density of retinal cones [36, 37]. The similarity between of our estimate of hyaena visual acuity (8.02 cpd; Table 1) and that estimated from study of retinal morphology (8.4 cpd, [36]) support our approach. This procedure would not, however, be appropriate for cheetahs (*Acinonyx jubatus)*, as they have increased cone densities and other adaptations that increase acuity relative to domestic cats [37, 38]. However, cheetahs hunt zebras rarely, so are of little relevance.

**Table 1:** Axial diameter (AD) of eyes used in generating species-specific contrast sensitivity functions, together with visual acuity estimates (cpd) of the four mammalian species of interest under different illumination conditions. Also shown is the corresponding minimum resolvable visual angle subtended on the retina by the target of interest. AD values for zebra and human (in parentheses) are provided for context, and were not used to estimate acuity.

	AD (mm)	AD Reference	Estimated Visual Acuity (cpd)			Minimum Resolvable Visual Angle (radians) x 10^4^		
Species			Photopic (daylight)	Mesopic (dusk; bright moonlight)	Scotopic (moonless night)	Photopic	Mesopic	Scotopic
Lion	41	[28]	13.42	7.66	1.89	6.503	11.39	46.17
Hyaena	24.8	[36]	8.02	4.60	1.17	10.88	18.97	74.59
Zebra	(41)	[30, 39]	23.30	12.46	1.61	3.745	7.004	54.20
Human	(24)	[40]	60.00	23.17	1.29	1.454	3.766	67.65

We followed a similar procedure to estimate the CSF of zebras. However, horses have considerably better acuity (cut-off ca. 23.3 cpd) than mammals of similar size (e.g., cows [30]). We therefore used the upper acuity limit of that reported for horses for zebra photopic acuity as their eyes are of similar size [39] and they are close relatives. This is based on the assumption that they share similar retinal topographies, one that should be validated by future anatomical studies. We set the mesopic acuity and CSF for zebras midway between the photopic and scotopic CSFs because this procedure closely estimates the human mesopic curve obtained from psychophysical experiments. Mesopic and scotopic acuities for lions and hyaenas, and the scotopic acuity for zebras, were scaled relative to that of the cat, under the assumption that differences in rod number would scale relative to eye size.

We use a linear systems approach to image filtering, which is based on the premise that an image can be represented equivalently in both the spatial and frequency domains, and that a linear transformation (e.g., a Fourier transform) between them preserves the identity of the original image. Here, a Fourier transform refers to a convolution procedure in which the spectra of natural world scenes were modified by the digital weighting functions (i.e., the CSFs) representative of the spatial vision abilities of a lion, spotted hyaena, zebra or human in high, intermediate or low light conditions.

Two-dimensional patterns can be represented by their Fourier spectrum—the linear sum of their component sine waves of specific spatial frequency, amplitude, phase and orientation. Since the CSF is assumed to represent the sensitivity of the visual system to these same components, it can be used as a “quasi-modulation transfer function” (MTF) through which an image’s components can be passed to arrive at a description of the source observer’s image pattern [41, 42]. In the forward transform, the frequency spectra of the image and a MTF or CSF are multiplied together. The resulting product is then inverse-transformed to yield a digitally filtered image representation of how it would appear after passing through a device-specific MTF (e.g., a lens system) or alternatively, when based on the CSF of a “design” observer [43].

In this study, the convolutions were performed by DK with a Vision and Aging Lab (VAL) custom CSF plug-in for ImageJ, a U.S. National Institutes of Health public domain Java-based image-processing program. The plug-in allows digital image spatial filtering with user-defined Butterworth, exponential or CSF filters. In this study, a Test Filter/Control Filter protocol was used wherein digital scenes were convolved using an observer-specific “test” photopic, mesopic or scotopic CSF filter in relation to a “gold standard” control filter–a young human adult photopic CSF [44]. This approach was used to facilitate presentation of the images for a visually healthy adult human observer in the conditions in which they would most likely be viewed, namely, photopic lighting. The only necessary exception to this approach was that the human photopic scenes filtered with the same CSF as both test and control filter would not modify the image. For this reason, human photopic scenes were filtered using a third-order Butterworth low-pass filter [45] for a visually healthy young adult with excellent acuity (i.e., a high frequency cut-off of 44 cpd, equivalent to 20/14 Snellen acuity).

Given the primary intent of our study was to examine visibility of largely achromatic zebra stripes in three lighting conditions, one of them achromatic (i.e., scotopic), to avoid potentially spurious exacerbation of luminance contrast differences we performed spatial filtering prior to the final colour adjustment of the images. Species-specific dichromatic colour vision filters were applied to the images by ADM to adjust for the effects of non-human species differences on spectral sensitivity and thus, for net luminance and chromatic contrast. We used a customizable Colour Vision Simulator software program [46], which allows us to set the peak sensitivity of the cone photopigments to adjust the chromaticity of the spatially filtered digital images to accord with known species-specific colour sensitivities. We used values of 430 nm for the peak sensitivity of the short-wave sensitive (blue) cones for all species, and 545 nm (zebra) and 553 nm (lion, hyaena) for long-wave sensitive cones [47–50]. Although this programme was developed to simulate variants of primate vision–as are commercially available alternatives (e.g., Vischeck)—and thus may introduce biases (e.g. non-primate mammals do not have a *macula lutea*), it does allow for the specification of cone sensitivities. Therefore, it provides the best available emulation of species-specific colour vision, and serves our general purpose of generating a better final representation of a scene experienced through the eyes of dichromatic viewers.

Finally, to estimate the impact of natural lighting changes on animal visibility, the mean bright-area luminance of each image was adjusted photometrically by DK to high, intermediate or low luminance within the photopic range using a Minolta LS-110 Spot Photometer. To maintain consistent luminance contrast while adjusting overall scene luminance, spot photometric measurements were carried out to find the mean contrast ratio of the highest on-screen luminance point on the animal relative to four immediately adjacent background locations (i.e., left, right, above and below) for the human observer images. The luminance of the brightest of the four points was then used as the reference for using the “brightness” control in Photoshop to find the highest (i.e., “photopic”) and lowest (“scotopic”) luminance levels at which the mean animal/background contrast was maintained (i.e., no luminance saturation). Due to natural variations in the images (i.e., scene elements, scene type and time of day) the scotopic-photopic luminance range of the reference location varied across scenes. The luminance of the reference point for mesopic images was set at one-third of the range above the scotopic level, and thus, two-thirds of the range below the photopic level. Each image was adjusted separately, with the same brightness increment/decrement applied to all four observer species.

### Vision in Dim Light

Non-primate mammals generally have better low-light sensitivity than humans due to the presence of a *tapetum lucidum*, a reflective “eye-shine” layer immediately behind the retina. Prior studies report that domestic cats have six times greater sensitivity to light than humans [51]. Additionally, the greater light-gathering ability of a larger eye (i.e., a bigger objective lens) benefits dim light vision. Thus, dark or dimly lit scenes viewed simultaneously by lions, zebras, and to a lesser extent spotted hyaenas, should appear brighter than they would to humans. Because of the limitations in the dynamic range of a computer screen or printed page, it is not possible to scale the brightness of our images to account for species-specific differences in sensitivity across photopic, mesopic and scotopic luminance conditions. However, because each animal will experience dark, dim and bright light levels in a relatively similar way over the 24-hour cycle, we believe that our approach provides a reasonable approximation of the relative conspicuity of stripes under changing illumination levels.

## Results

### Relative Conspicuity of Zebra Stripes to Lions, Hyaenas, Zebras and Humans

**Fig 1:**
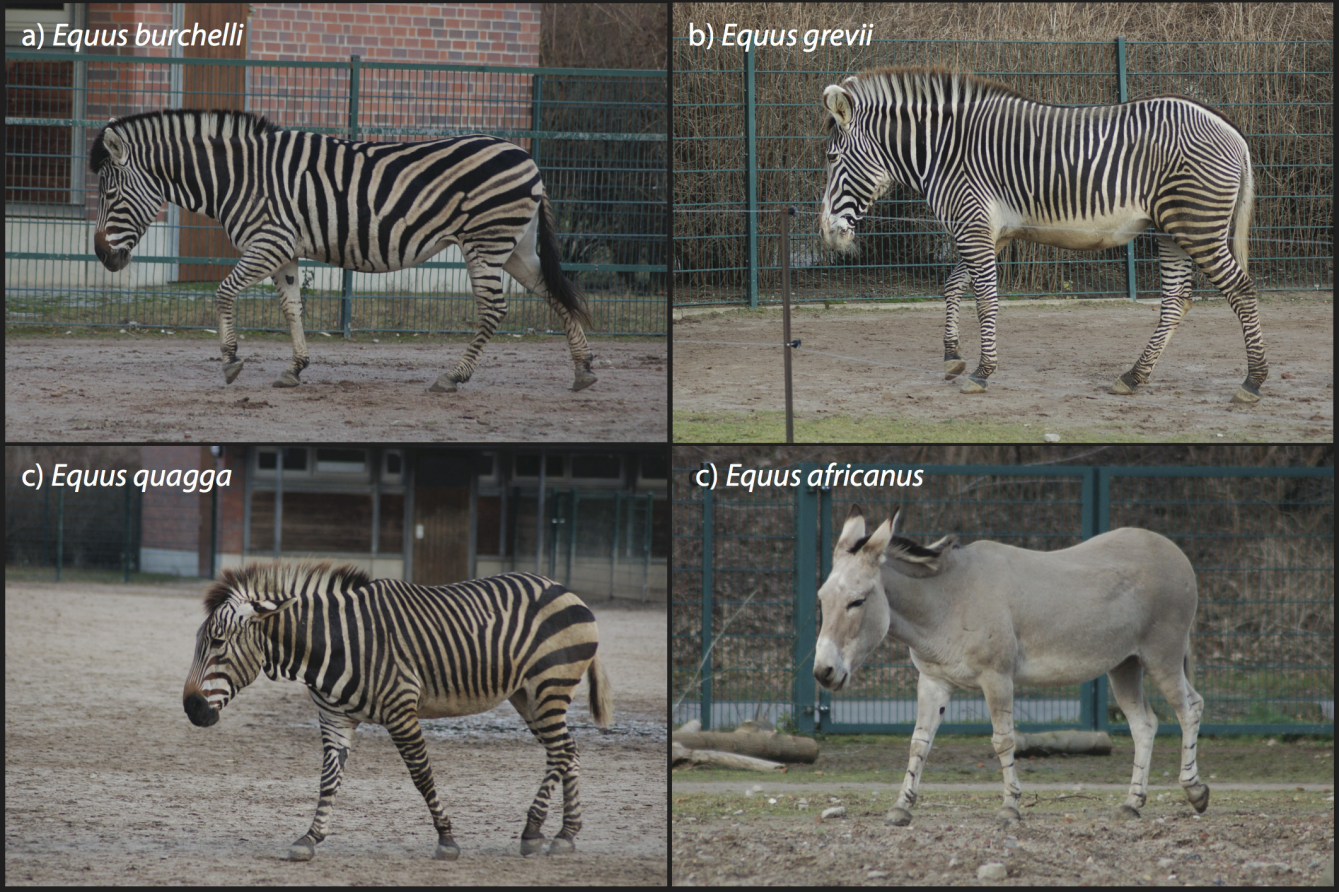
Photographs of a (a) plains, (b) mountain, and (c) Grevy’s zebra, and (d) African wild ass in the Tierpark Zoo, Berlin. All photos by Tim Caro.

The body location of the widest stripes varies among zebra species. The widest stripes are commonly on the flank of the plains zebra, the neck of the Grevy’s zebra and the rump of the mountain zebra (Table 2; S2 Dataset). The thinnest stripes for all three species are consistently on the forelimbs and range from about 1/3^rd^ the width of the widest stripes (Grevy’s) to 1/5^th^ the width of the widest stripes found in mountain zebras. Stripes may also be found on the legs of other congeners (e.g., *Equus africanus)* (Fig 1).

**Table 2:** Estimated maximum distance at which zebra stripes can be resolved by different species of viewers, by body region.

				Human			Lion			Hyaena			Zebra		
Species of zebra	Relative size of stripes	Position	Ave width (cm)	Daylight (photopic)	Dusk (mesopic)	Night (scotopic)	Daylight (photopic)	Dusk (mesopic)	Night (scotopic)	Daylight (photopic)	Dusk (mesopic)	Night (scotopic)	Daylight (photopic)	Dusk (mesopic)	Night (scotopic)
Plains (5)	widest	Side	5.23	359.59	138.86	7.73	80.43	45.91	11.33	48.06	27.57	7.01	139.64	74.67	9.65
	narrowest	forelimb	1.19	81.82	31.60	1.76	18.30	10.45	2.58	10.94	6.27	1.60	31.77	16.99	2.20
Grevy's (3)	widest	neck	2.83	194.58	75.14	4.18	43.52	24.84	6.13	26.01	14.92	3.79	75.56	40.41	5.22
	narrowest	forelimb	1.00	68.75	26.55	1.48	15.38	8.78	2.17	9.19	5.27	1.34	26.70	14.28	1.84
Mountain (1)	widest	rump	6.38	438.66	169.39	9.43	98.11	56.00	13.82	58.63	33.63	8.55	170.34	91.09	11.77
	narrowest	forelimb	1.41	96.94	37.44	2.08	21.68	12.38	3.05	12.96	7.43	1.89	37.65	20.13	2.60

Under photopic (daylight) conditions, humans with the best acuity possible (20/10) should be able resolve the widest stripes of our main species of interest–a plains zebra–at distances up to 360 m. A human with good average (i.e., 20/20) acuity would resolve stripes at half this distance (180m). The limit of a lion’s and spotted hyaena’s ability to resolve these same stripes under photopic (daylight) conditions is projected to be at distances of approximately 80 m and 48 m respectively, whereas other zebras might resolve conspecific stripes up to 140 m away. The maximum distance at which the widest stripes could be resolved is slightly extended for the mountain zebra (ranging from 439 m to 59 m–from best (human), to worst (hyaena))–and is much less for the Grevy’s zebra (195 m–26 m), which has the thinnest stripes (Table 2; Fig 1).

**Fig 2:**
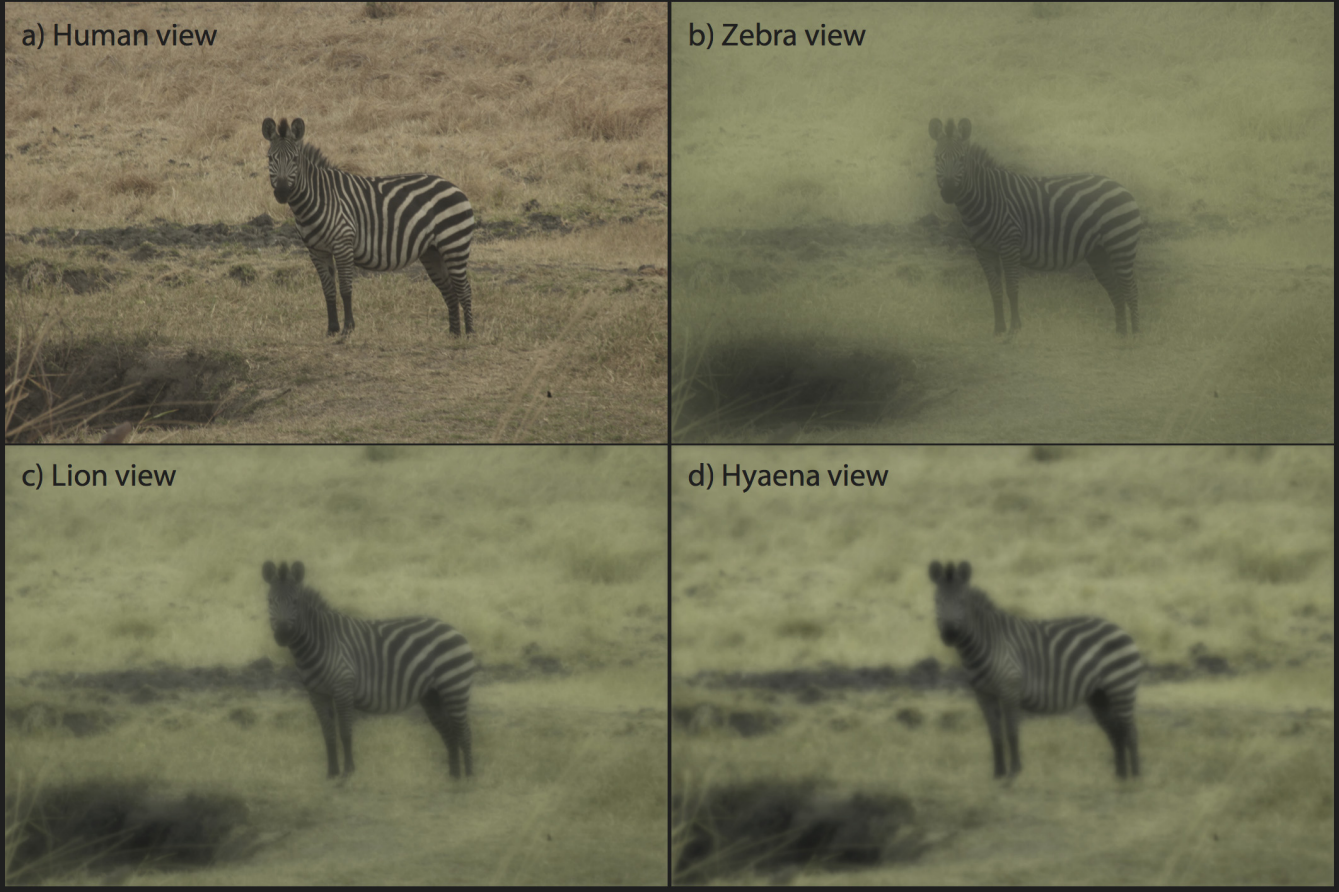
Image of a solitary plains zebra at a distance of 6.4 m as it may appear to a human, zebra, lion and spotted hyaena under photopic conditions. As shown, stripes are detectable to all species at this distance.

The maximum distance of resolution decreases for areas of the body with thinner stripes. For example, the forelimbs of a plains zebra may appear to be uniform grey at distances of 82 m (humans), 18 m (lions), 11 m (hyaenas), and 32 m (zebras; Table 2). These values are based on measurements of adult skins. Neonate zebras have thinner (and often brownish stripes of lower contrast) so their stripes would disappear at even shorter distances. We simulated the adjusted spatial and colour profiles of a solitary zebra seen at close range (6m away, Fig 2) as they might appear to our four observer species under photopic conditions. We additionally simulated the appearance of a small group of zebras from 16 m away and a large group of zebras in the distance (closest animals are at 111 m) for all targeted observers. These images demonstrate how stripe resolution is hindered as distance increases and is inversely related to acuity.

**Fig 3:**
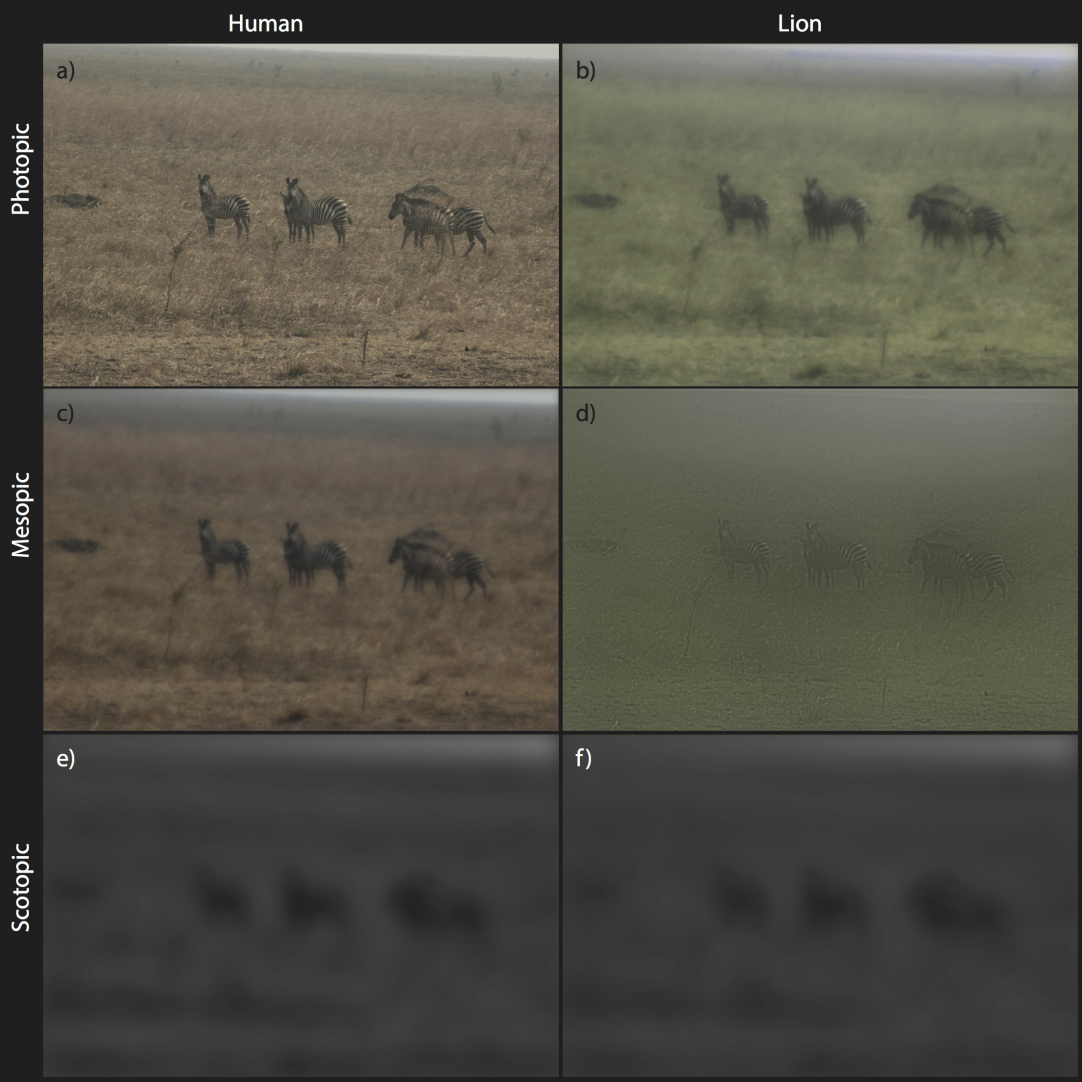
A small group of plains zebra taken at a real-world equivalent of 16.4 m as they may appear to a human (a,c,e) and lion (b,d,f) under photopic (bright; daylight), mesopic (dim; dusk) and scotopic (dark; moonless night) conditions. Stripe visibility falls off from human vision to lion vision and as ambient light decreases.

Projected acuity declines with light level (Table 1). Under the mesopic (dawn/dusk) levels used in this study, the distance at which the widest plains zebra stripes are resolvable by humans drops to 139 m which is 38% of the maximum photopic range (equivalent to a reduction from 20/10 to about 20/26). Similar–but less drastic–decreases occur for the visual systems of other species (75 m zebras, 46 m lions, 26 m hyaenas), although, importantly, humans outperform them by a wide margin. Contrast sensitivity functions and acuities of all four observer species are most similar under scotopic conditions (S1 Fig; Table 1). Based on their larger eye size, zebras and lions are expected to have slightly better acuity than humans in dim light, although this needs to be verified by physiological studies examining the rod densities, and degree of retinal spatial summation. Stripes can only be resolved at very close distances under scotopic conditions (lions 11 m, hyaenas 7 m, zebras 10 m, and humans 8 m, Table 2). In Fig 3 we demonstrate the effects of decreasing ambient light by presenting the same image of a small group zebras at 16 m to approximate their appearance to humans and lions under “photopic” (a,b), “mesopic” (c,d), and “scotopic” (e,f) conditions. These images align well with our calculations (Table 2) that humans and lions can resolve the widest stripes of zebras under mesopic conditions, but cannot resolve stripes under scotopic conditions at distances beyond 16.4 m.

### Relative Conspicuity of Other Prey Species

**Fig 4:**
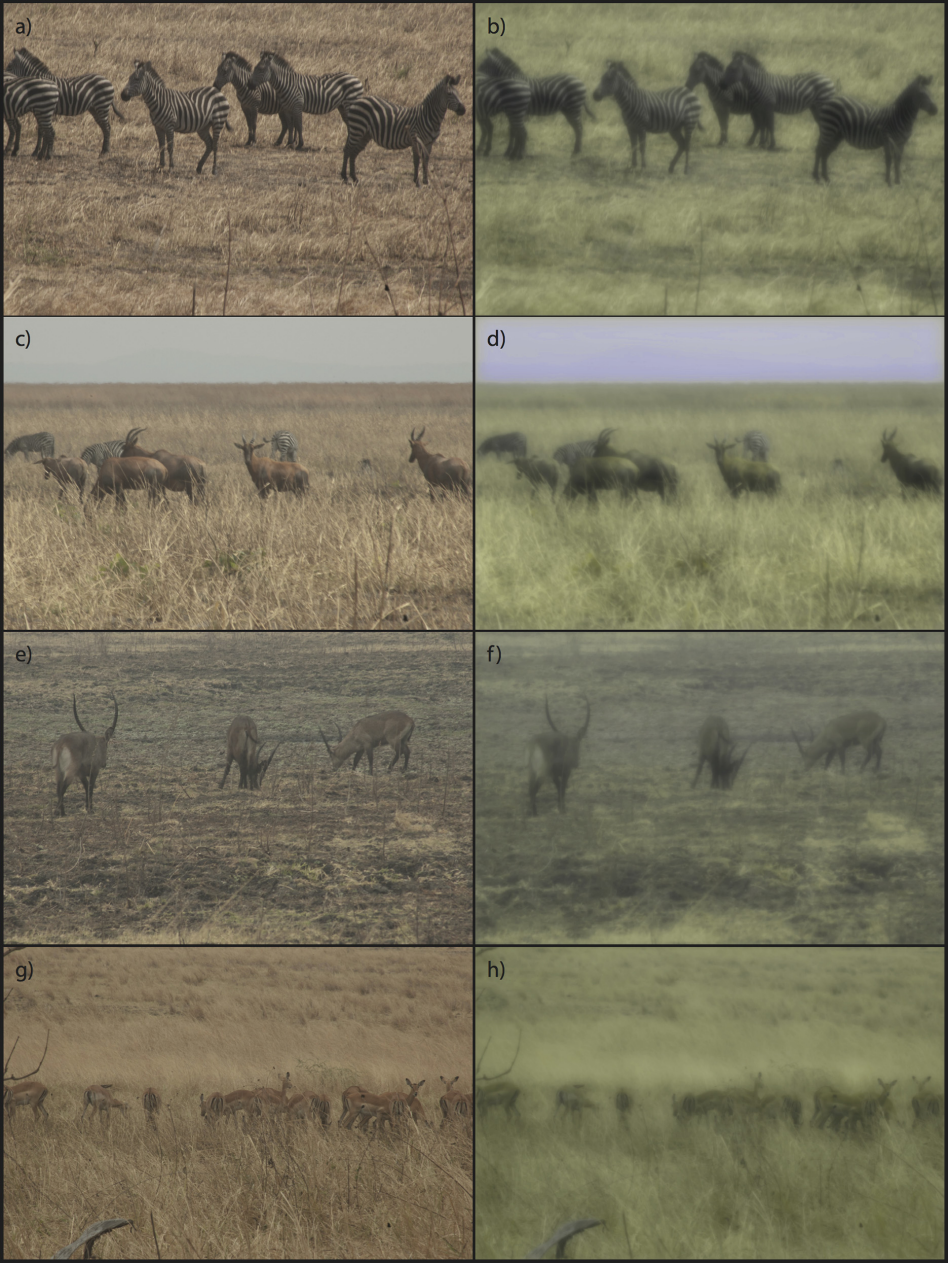
Groups of zebra (a,b), zebra and topi together (c,d), waterbuck (e,f), and impala (g,h) on the Katavi plains from a real-world viewing distance of 9 m. Images are scaled such that size variation between species reflects true differences among species. Images are modeled for human (left panels) and lion (right panels) visual systems under photopic conditions.

A separate approach to determining whether stripes aid in concealment is to compare images of striped and unstriped prey species modeled for a human versus a lion (Fig 4). If striping is a form of crypsis, zebras should be more difficult to see through a predator’s eye than a uniformly coloured herbivore of similar size and space-averaged luminance. As shown in the top set of panels, stripes on individual zebras do not make zebras blend together in simulated lion vision; individuals are still clearly discriminable. In the pair of panels second from the top, even the more distant zebras are easy to discern. Comparison of the top three panels indicates that waterbuck, topi and zebras are all relatively easy to see. Finally, from a lion’s point of view, the smaller impalas are subjectively more difficult to detect in the tall grass present in the scene.

To add greater objectivity to our conclusions, CH applied a Sobel edge-detection algorithm to these images. This biologically-relevant analysis is well suited to detecting naturally-occurring features, including body contours and pelage patterns. We set a detection threshold—which determines the cut-off intensity of edges displayed relative to maximum intensity of the image—of 0.15. Adjustment of the threshold to 0.3 decreases the detail rendered but does not alter the result. This analysis confirmed that outlines of animals are readily distinguishable from the background scene (S2 Fig). Finally, the hue-matching property of brownish species and grasses in the dry season likely provides better camouflage during daylight than does the greyish space-averaged luminance of zebra stripes, an advantage that would disappear in scotopic conditions.

### Conspicuity in Woodland

**Fig 5:**
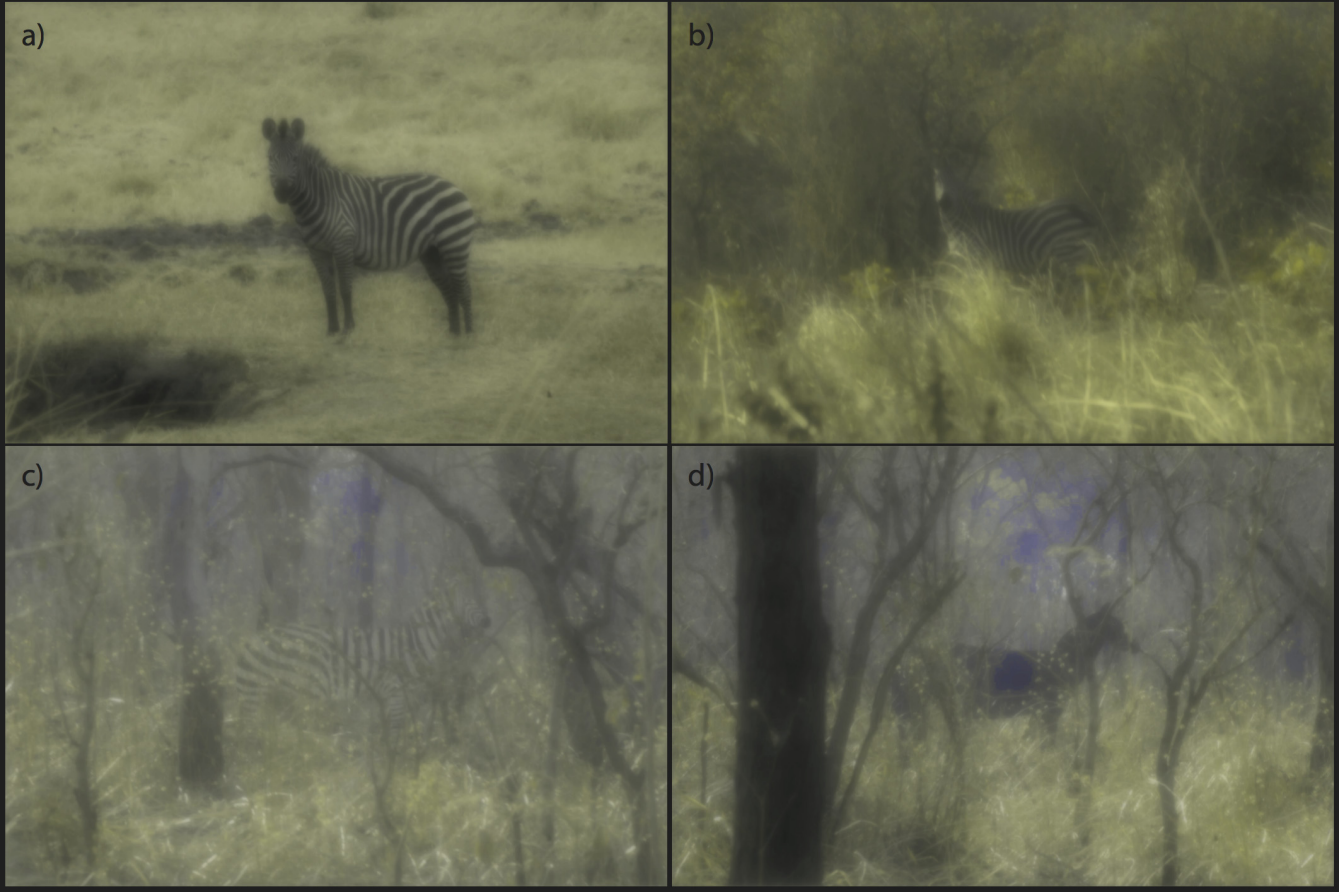
A solitary zebra on the plains (a), and on the outskirts of a forest (b), as well as striped (c) and uniformly grey (d) models of zebras in the woodlands. Images are modeled for a lion visual system under photopic conditions.

To assess the conspicuity of zebra stripes amidst a background of trees, we compared photographs of live zebras and also life-sized models of a striped zebra with a hypothetical solid grey zebra in woodland. A solitary zebra appears less conspicuous in a forested setting than on the open plains, and a striped zebra model was less conspicuous than a solid grey model in woodland to all visual systems simulated (Fig 5). This subjective interpretation is reinforced by the results of the Sobel edge-detection algorithm, which was much better at detecting body contour on the plains than in woodland scenes (S3 Fig). This effect may in part be due to increased luminance contrast outside of wooded areas: measures of both live zebras in captivity and photographs of wild zebras reveal greater luminance contrast between animals and unobstructed sky–a backdrop on the plains–than between animals and structurally complex vegetative backdrops such as that found in woodland. Arguably, the decreased conspicuity of the zebra in a woodland setting can be at least partially explained by the resemblance of black stripes to the vertical outlines of dark tree branches and brush, rather than to disruptive colouration breaking up the body outline (i.e., disruptive colouration). In contrast, a solid grey object of like size and shape has fewer large dark object counterparts in the woodland environment.

## Discussion

We combine field data, digital images and psychophysical approaches to address the long-standing crypsis hypothesis for the function of zebra stripes by modeling their perception by chief predators, conspecifics and humans. Our analyses indicate that, under daylight conditions, humans can resolve zebra stripes at considerably greater distances than can large carnivores or zebras, but that all four observer types perform in a roughly equivalent fashion under scotopic conditions; (S1 Table). Under photopic conditions, however, humans can resolve stripes at distances 2.6 times greater than that of zebras, and at 4.5 and 7.5 times the distances of lions and spotted hyaenas, respectively. Wide stripes appear to be discernible to lions at 44 m or closer (Grevy’s) and from 98 m or less (mountain zebras), and to spotted hyaenas, at 59 m or less (Table 2).

Stripe visibility decreases dramatically as light falls. At dusk, when hunting by carnivores normally begins, humans can resolve stripes from greater distances than other mammals: three times those of lions, five times further than spotted hyaenas, and 1.9 times more distant than zebras (S1 Table). Under such mesopic conditions, we project that lions can discern stripes at 25–56 m and hyaenas at 15–34 m, but beyond these distances zebras’ bodies simply appear grey. Under scotopic conditions, the four species have similar spatial vision and so need to be very close to see stripes. For a lion, it can resolve the widest stripes at only 6–14 m, and a spotted hyaena at 4–9 m.

These findings suggest strongly that stripes themselves are unlikely to be a form of crypsis at far distances because predators would not be able to discern the black stripes against a treed background, or see white stripes blending in with bright shafts of light between trees (background matching). Nor by the same reasoning could striping operate by disrupting the zebra’s body outline. At dusk or during bright moonlight (mesopic conditions bright enough to support mammalian cone function [52, 53]) the resolution distances decrease even further. Thus, stripes cannot help zebras blend in with the background except when a zebra is close to a predator, distances at which predators could likely smell or hear zebras moving or breathing as they are particularly noisy herbivores [54]. A limitation of our study is that the projected detection ranges are necessarily based on estimated CSFs, due to the impracticality of determining CSFs behaviourally for these species. However, it is unlikely that refinements of detection ranges based on true CSFs would drastically alter our conclusions, especially for the predators—lions and hyaenas—for which retinal topography data indicate low acuity. Humans and other anthropoid primates have anomalously acute vision, especially under the daylight conditions when humans strongly centre our activities and observations. Here we take an important stride to see zebras through the eyes of predators and conspecifics; our results have important implications for hypotheses on the evolution of conspicuous pelage patterning.

### Implications for the Evolution of Zebra Striping

Since the chief predators of zebras cannot resolve distant stripes, early arguments concerning zebras being cryptic as advanced by Galton and others, arguments that have persisted for more than a century, are contradicted by our analyses. Furthermore, comparisons between uniformly coloured species and zebras (Fig 5) show that all species are conspicuous on the plains because their body silhouette is high in luminance contrast relative to the sky. These preliminary data suggest that in open plains environments, a setting where zebras spend much of the year, stripes do not disrupt the outline of the body [1] and so are unable to confer cryptic advantages compared to unstriped species. In some wooded environments, where stripes might resemble branches and tree trunks of saplings (although not large trunks), again stripes can only be resolved by nearby predators; zebras appear grey under other conditions. It is worth noting that zebras might be even noisier in woodland than grassland habitats because of twigs and branches underhoof and so the chances of zebra prey remaining hidden when near to a predator are likely minimal. Only a stationary, silent nearby zebra in a woodland habitat would benefit from crypsis due to stripes, a distance at which scent could be a cue to predators. In short, our data fail to support the hypothesis that stripes confer a form of crypsis against predators under a variety of conditions. More generally, the likelihood that stripes reduce predation, by lions at least, is improbable given that zebras are a preferred prey of lions in most parts of Africa [55].

In this study we were also able to estimate how zebras see stripes. Assuming that zebra spatial vision is similar to that of horses, zebras can likely see stripes from considerably greater distances than can their predators, although less well than humans. We therefore cannot reject the hypothesis that stripes may assist recognition of conspecifics or individuals, although stripes promoting species recognition seem improbable given the limited extent of allopatry in the three species of zebra. Field observations do not support the idea of stripes enhancing allogrooming, social bonding, individual recognition or being an indicator of phenotypic quality or health [54]. Nor is striping related to crude categories of social organization, namely harem defense polygyny or to resource defense across equids where social requirements might differ. Finally, domestic horses are capable of sophisticated individual recognition using visual cues in the absence of stripes [56] and so it seems somewhat implausible that their close relative, the zebra, needs stripes to do this.

In sum, our results do not lend support to stripes being a form of anti-predator crypsis, leading us to reject this longstanding hypothesis on the adaptive significance of zebra striping. Zebra stripes have also been hypothesized to reduce predation through aposematism [2, 54]. Our results suggest this too is unlikely: during periods of peak hunting lions and hyaenas can respectively discern stripes from less than 56 m and 34 m away, distances at which they have likely already committed themselves to attack. While our analyses suggest that zebras can likely discern stripes at greater distances than the carnivores that prey on them, it does not mean that striping is driven by social necessity, as un-striped congeners are highly social and able to recognize individuals in the absence of striped pelage. Explanations for zebra striping must be sought elsewhere; indeed recent analyses have shown that striping in equids is associated with tabanid biting fly annoyance [57], and furthermore that avoidance of such parasitism is the most parsimonious explanation for striping in zebras [58].

## Supporting Information

S1 Dataset Luminance (cd/m^2^) and illuminance (lux) data of zebra pelage, background environment, and ambient lighting taken at the Calgary Zoo. (CSV)

S2 Dataset Original zebra stripe width data collected for this study. (CSV)

S1 Fig Contrast sensitivity functions (CSFs) estimated for lions, hyaenas, zebras and humans under (a) photopic, (b) mesopic and (c) scotopic conditions. The CSF for a domestic cat is illustrated for reference.
(TIFF)

S2 Fig Digital images, following application of Sobel edge-detection algorithm, of (a) groups of zebra, (b) zebra and topi together, (c) waterbuck, and (d) impala on the Katavi plains from a real-world viewing distance of 9 m under photopic conditions. Images are modeled under a 0.30 threshold (left panels) and 0.15 threshold (right panels) for a lion’s visual system.
(TIFF)

S3 Fig Digital images, following application of Sobel edge-detection algorithm, of a solitary zebra on (a) the plains and (b) the outskirts of a forest, along with models of (c) striped and (d) uniformly grey zebras in the woodlands. Images are modeled for a lion visual system under photopic conditions.
(TIFF)

S1 Table Relative visual performance of different species under decreasing ambient light. Values represent how much closer to the target the species listed vertically would need to be to resolve the same level of spatial detail as the species listed horizontally.
(DOCX)

## References

[1] Thayer G (1909) Concealing-colouration in the animal kingdom: an exposition of the laws of disguise through colour and pattern: Being a summary of Abbott H. Thayer’s discoveries New York: Macmillan.

[2] Matthews LH (1971) The life of mammals New York: Universe books.

[3] Stevens M, Yule D, Ruxton G (2008) Dazzle colouration and prey movement. Proc Roy Soc Lond B 275: 2639–43. 10.1098/rspb.2008.0877 PMC2605810 18700203

[4] Kingdon J (1984) The zebra’s stripes: an aid to group cohesion In: Macdonald D, editor. The encyclopedia of mammals. Oxford: Equinox p. 486–7.

[5] Waage JK (1981) How the zebra got its stripes-biting flies as selective agents in the evolution of zebra coloration. J Entomol Soc South Africa. 44: 351–8.

[6] Egri A, Blaho M, Kriska G, Farkas R, Gyurkovszky M, Akesson S, et al (2012) Polarotactic tabanids find striped patterns with brightness and/or polarization modulation least attractive: an advantage of zebra stripes. J Exp Biol. 215: 736–45. 10.1242/jeb.065540 22323196

[7] Morris D (1990) Animal watching A field guide to animal behaviour. London: Johnathan Cape.

[8] Wallace AR (1896) Darwinism: an exposition of the theory of natural selection with some of its applications London: Macmillan Co.

[9] Darwin CR (1906) The descent of man, and selection in relation to sex 2nd edition London: John Murray.

[10] Godfrey D, Lythgoe JN, Rumball DA (1987) Zebra stripes and tiger stripes: the spatial frequency distribution of the pattern compared to that of the background is significant in display and crypsis. Biol J Linn Soc 32: 427–33.

[11] Uhlrich DJ, Essock EA, Lehmkuhle S (1981) Cross-species correspondence of spatial contrast sensitivity functions. Behav Brain Res. 2: 291–9. 6784738 10.1016/0166-4328(81)90013-9

[12] Kruuk H (1972) The spotted hyena Chicago: University of Chicago Press.

[13] Schaller GB (1972) The serengeti lion Chicago: University of Chicago Press.

[14] Ginsburg A, Evans D, Sekuler R, Harp S (1982) Contrast sensitivity predicts pilots' performance in aircraft simulators. Am J of Optom Physiol Opt. 59: 105–9. 7055195 10.1097/00006324-198201000-00020

[15] Evans DW, Ginsburg AP (1985) Contrast sensitivity predicts age-related differences in highway-sign discriminability. Hum Factors. 27: 637–42. 3833637 10.1177/001872088502700602

[16] Owsley C, Sloane ME (1987) Contrast sensitivity, acuity, and the perception of "real world" targets. Br J Ophthalmol. 71: 791–6. 3676151 10.1136/bjo.71.10.791 PMC1041308

[17] Shapley R (2009) Linear and nonlinear systems analysis of the visual system: why does it seem so linear? A review dedicated to the memory of Henk Spekreijse. Vision Res. 49: 907–21. 10.1016/j.visres.2008.09.026 18940193 PMC2705991

[18] Ginsburg AP. Visual information processing based on spatial filters constrained by biological data Springfield, VA: National Technical Information Service, 1978.

[19] Caro T (2013) The colours of extant mammals. Semin Cell Dev Biol. 24: 542–52. 10.1016/j.semcdb.2013.03.016 23567208

[20] Selous FC (1908) African nature notes and reminiscences London: Macmillan and Co Ltd.

[21] Blake R, Cool SJ, Crawford MLJ (1974) Visual resolution in the cat. Vision Res. 14: 1211–7. 4428628 10.1016/0042-6989(74)90218-1

[22] Birch D, Jacobs GH (1979) Spatial contrast sensitivity in albino and pigmented rats. Vision Res. 19: 933–7. 516464 10.1016/0042-6989(79)90029-4

[23] Pasternak T, Merigan W (1981) The luminance dependence in the cat of spatial vision. Vision Res. 21: 1333–9. 7314518 10.1016/0042-6989(81)90240-6

[24] Stevens M, Párraga CA, Cuthill IC, Partridge JC, Troscianko TS (2007) Using digital photography to study animal coloration. Biol J Linn Soc. 90: 211–37.

[25] Keller J, Strasburger H, Cerutti DT, Sabel BA (2000) Assessing spatial vision—automated measurement of the contrast-sensitivity function in the hooded rat. J Neurosci Meth. 97: 103–10. 10.1016/s0165-0270(00)00173-4 10788664

[26] Hanke FD, Scholtyssek C, Hanke W, Dehnhardt G (2011) Contrast sensitivity in a harbor seal (*Phoca vitulina*). J Comp Physiol A. 197: 203–10. 10.1007/s00359-010-0600-y 20981455

[27] Pelli DG (1985) Uncertainty explains many aspects of visual contrast detection and discrimination. J Opt Soc Am A. 2: 1508–32. 4045584 10.1364/josaa.2.001508

[28] Howland HC, Merola S, Basarab JR (2004) The allometry and scaling of the size of vertebrate eyes. Vision Res. 44: 2043–65. 15149837 10.1016/j.visres.2004.03.023

[29] Kemp A, Kirk EC (2014) Eye size and visual acuity influence vestibular anatomy in mammals. Anat Rec. 297: 781–90. 10.1002/ar.22892 24591307

[30] Veilleux CC, Kirk EC (2014) Visual acuity in mammals: effects of eye size and ecology. Brain Behav Evol. 83: 43–53. 10.1159/000357830 24603494

[31] De Silva Souza G, Gomes B, Silveira LC (2011) Comparative neurophysiology of spatial luminance contrast sensitivity. Psychol Neurosci. 4: 29–48.

[32] Muir DW MD (1973) Visual resolution and experience: acuity deficits in cats following early selective visual deprivation. Science. 180: 420–2. 4700602 10.1126/science.180.4084.420

[33] Bisti S, Maffei L (1974) Behavioural contrast sensitivity of the cat in various visual meridians. J Physiol. 241: 201–10. 4425345 10.1113/jphysiol.1974.sp010649 PMC1331081

[34] Jacobsen SG, Franklin KBJ, Mcdonald WI (1976) Visual acuity of the cat. Vision Res. 16: 1141–3. 969226 10.1016/0042-6989(76)90254-6

[35] Blake R (1988) Cat spatial vision. Trends Neurosci. 11: 78–83. 2465604 10.1016/0166-2236(88)90169-5

[36] Calderone JB, Reese BE, Jacobs GH (2003) Topography of photoreceptors and retinal ganglion cells in the spotted hyena (*Crocuta crocuta*). Brain Behav Evol. 62: 182–92. 14573992 10.1159/000073270

[37] Ahnelt PK, Schubert C, Kubber-Heiss A, Anger E (2006) Adaptive design in retinal cone topographies of the domestic cat, cheetah and other felids. 6th Scientific Meeting of the European Association of Zoo and Wildlife Veterinarians (EAZWV) Budapest, Hungary.

[38] Ahnelt PK, Schubert C, Kubber-Heiss A, Schiviz A, Anger E (2006) Independent variation of retinal S and M cone photoreceptor topographies: A survey of four families of mammals. Visual Neurosci. 23: 429–35. 10.1017/S095252380623342X 16961976

[39] Kirk EC (2006) Eye morphology in cathemeral lemurids and other mammals. Folia Primatol. 77: 27–49. 16415576 10.1159/000089694

[40] Schwartz SH, Meese T (2010) Visual perception: A clinical orientation New York, NY: McGraw-Hill Medical Publishing Division.

[41] De Valois RL, De Valois KK (1988) Spatial Vision. Oxford: Oxford University Press.

[42] Michael R, Guevara O, de la Paz M, Alvarez de Toledo J, Barraquer RI (2011) Neural contrast sensitivity calculated from measured total contrast sensitivity and modulation transfer function. Acta Ophthalmol. 89: 278–83. 10.1111/j.1755-3768.2009.01665.x 19909292

[43] Daugman JG, Kammen DM (1986) Pure orientation filtering: A scale-invariant image-processing tool for perception research and data compression. Behav Res Meth Ins C. 18: 559–64.

[44] Owsley C, Sekuler R, Siemsen D (1983) Contrast sensitivity through adulthood. Vision Res. 23: 689–99. 6613011 10.1016/0042-6989(83)90210-9

[45] Kwon M, Legge GE (2012) Spatial-frequency requirements for reading revisited. Vision Res. 62: 1–10. 22521659 10.1016/j.visres.2012.03.025 PMC3653576

[46] Melin AD, Kline DW, Hickey C, Fedigan LM (2013) Food search through the eyes of a monkey: a functional substitution approach for assessing the ecology of primate color vision. Vision Res. 86: 87–96. 10.1016/j.visres.2013.04.013 23643907

[47] Jacobs GH (1993) Distribution and nature of colour vision among the mammals. Biol Rev. 68: 413–71. 8347768 10.1111/j.1469-185x.1993.tb00738.x

[48] Yokoyama S, Radlwimmer FB (1999) The molecular genetics of red and green color vision in mammals. Genetics. 153: 919–32. 10511567 10.1093/genetics/153.2.919 PMC1460773

[49] Carroll J, Murphy CJ, Neitz M, Ver Hoeve JN, Neitz J (2001) Photopigment basis for dichromatic colour vision in the horse. J Vision. 1: 80–7. 10.1167/1.2.2 12678603

[50] Jacobs GH (2009) Evolution of colour vision in mammals. Philos Trans R Soc Lond B Biol Sci. 364: 2957–67. 10.1098/rstb.2009.0039 19720656 PMC2781854

[51] Warrant E (2014) Vision in Dim Light In: Cronin TW, Johnsen S, Marshall J, Warrant E, editors. Visual Ecology. Princeton, NJ: Princeton University Press p. 262–87.

[52] Roth LS, Balkenius A, Kelber A (2008) The absolute threshold of colour vision in the horse. PLoS One. 3: e3711 10.1371/journal.pone.0003711 19002261 PMC2577923

[53] Melin AD, Moritz GL, Fosbury RAE, Kawamura S, Dominy NJ (2012) Why aye-ayes see blue. Am J Primatol. 74: 185–92. 24006536 10.1002/ajp.21996

[54] Caro T (In Press) Zebra stripes Chicago: University of Chicago Press.

[55] Hayward M, Kerley G (2005) Prey preferences of the lion (*Panthera leo*). J Zool Lond. 267: 309–22.

[56] Proops L, McComb K, Reby D (2009) Cross-modal individual recognition in domestic horses (*Equus caballus*). Proc Nat Acad Sci. 106: 947–951. 10.1073/pnas.0809127105 19075246 PMC2630083

[57] Caro T, Izzo A, Reiner RC Jr., Walker H, Stankowich T (2014) The function of zebra stripes. Nat Commun. 5: 3535 10.1038/ncomms4535 24691390

[58] Caro T, Stankowich T (2015) Concordance on zebra stripes: a comment on Larison et al. 2015. Roy Soc Open Sci. 2: 150323. 26473053 10.1098/rsos.150323 PMC4593687

