# Correction: Zebra Stripes through the Eyes of Their Predators, Zebras, and Humans

**DOI:** 10.1371/journal.pone.0151660

**Published:** 2016-03-17

**Authors:** Amanda D. Melin, Donald W. Kline, Chihiro Hiramatsu, Tim Caro

Fig 1 and its legend appear incorrectly in the published article. Please see the correct Fig 1 and its legend here.

**Fig 1:**
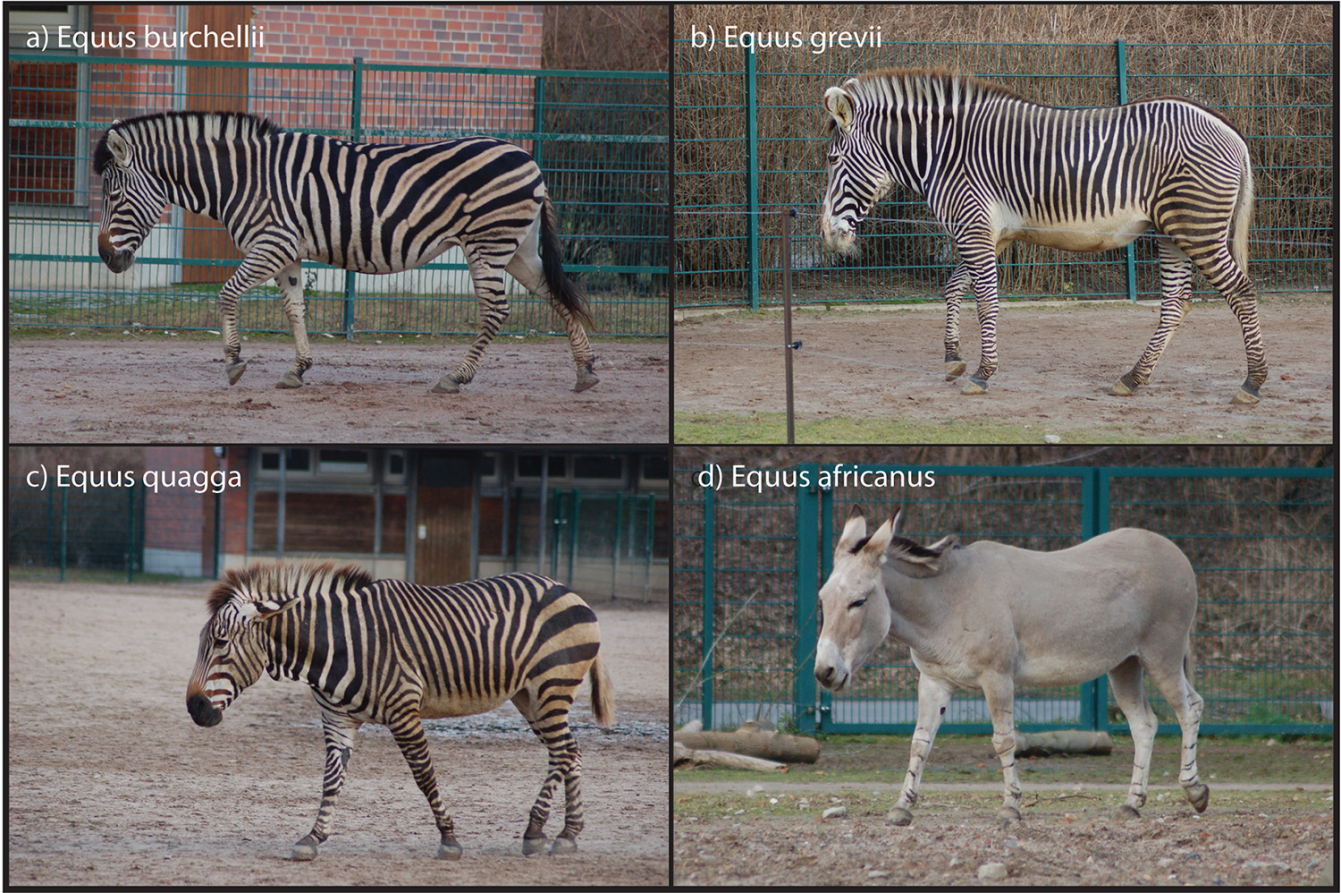
Photographs of (a) Plains zebra, (b) Grevy’s zebra, (c) Mountain zebra, and (d) African wild ass in the Tierpark Zoo, Berlin. All photos by Tim Caro.

The URL reference in the penultimate sentence of the Materials and Methods section under the heading “Collection of Digital Photographs” is incorrect. The correct sentence is: A complete dataset of all original and filtered images is available at the Harvard Dataverse, URL: http://dx.doi.org/10.7910/DVN/OHWWNR.
