# Supplementary figures and images for: Zebra Stripes through the Eyes of Their Predators, Zebras, and Humans
Source: PLoS One. 2016 Jan 22;11(1):e0145679. doi: 10.1371/journal.pone.0145679 (PMC4723339; doi:10.1371/journal.pone.0145679)

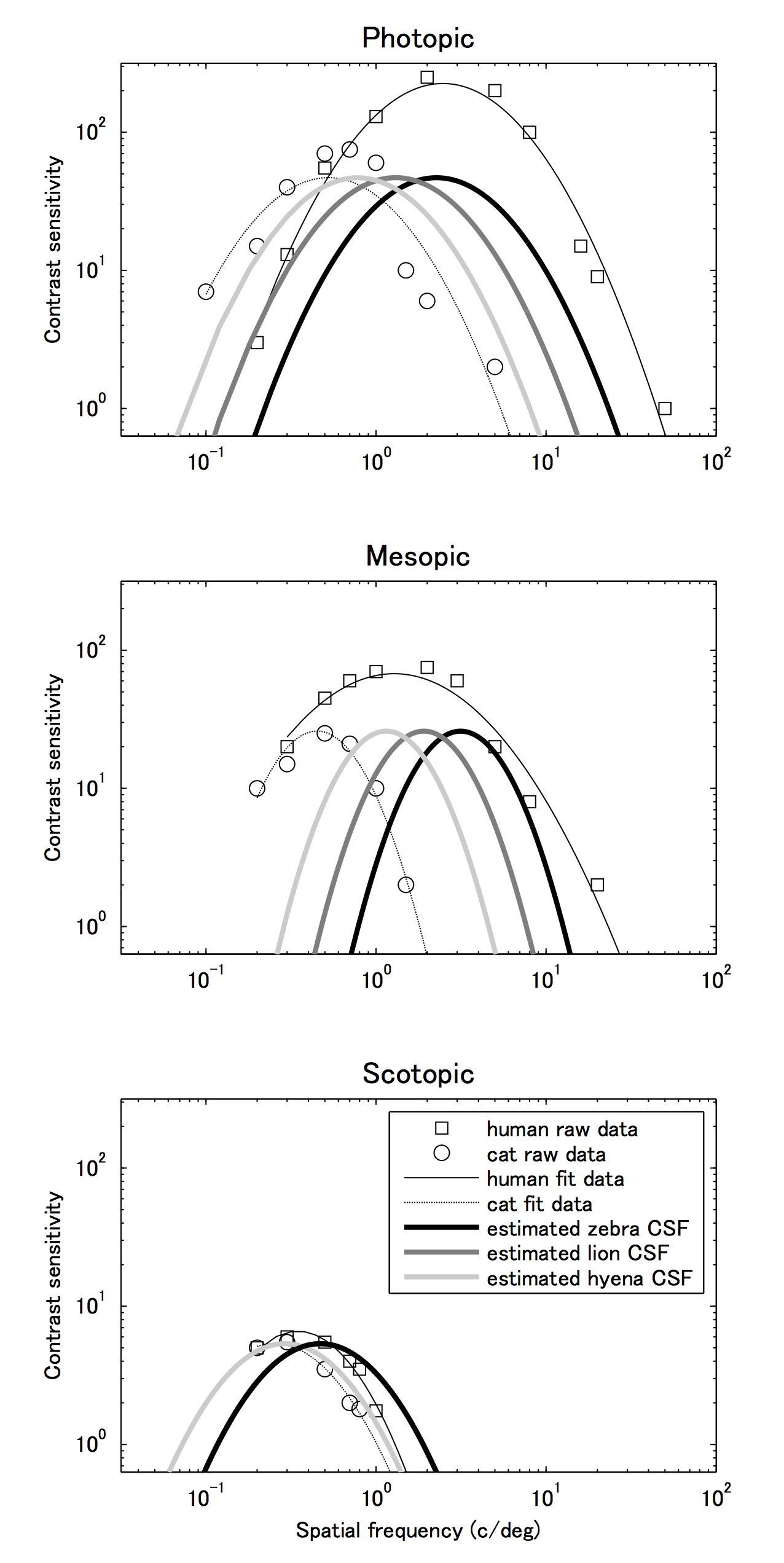

Supplement: S1 Fig — The CSF for a domestic cat is illustrated for reference. (TIFF) [file pone.0145679.s003.tiff]

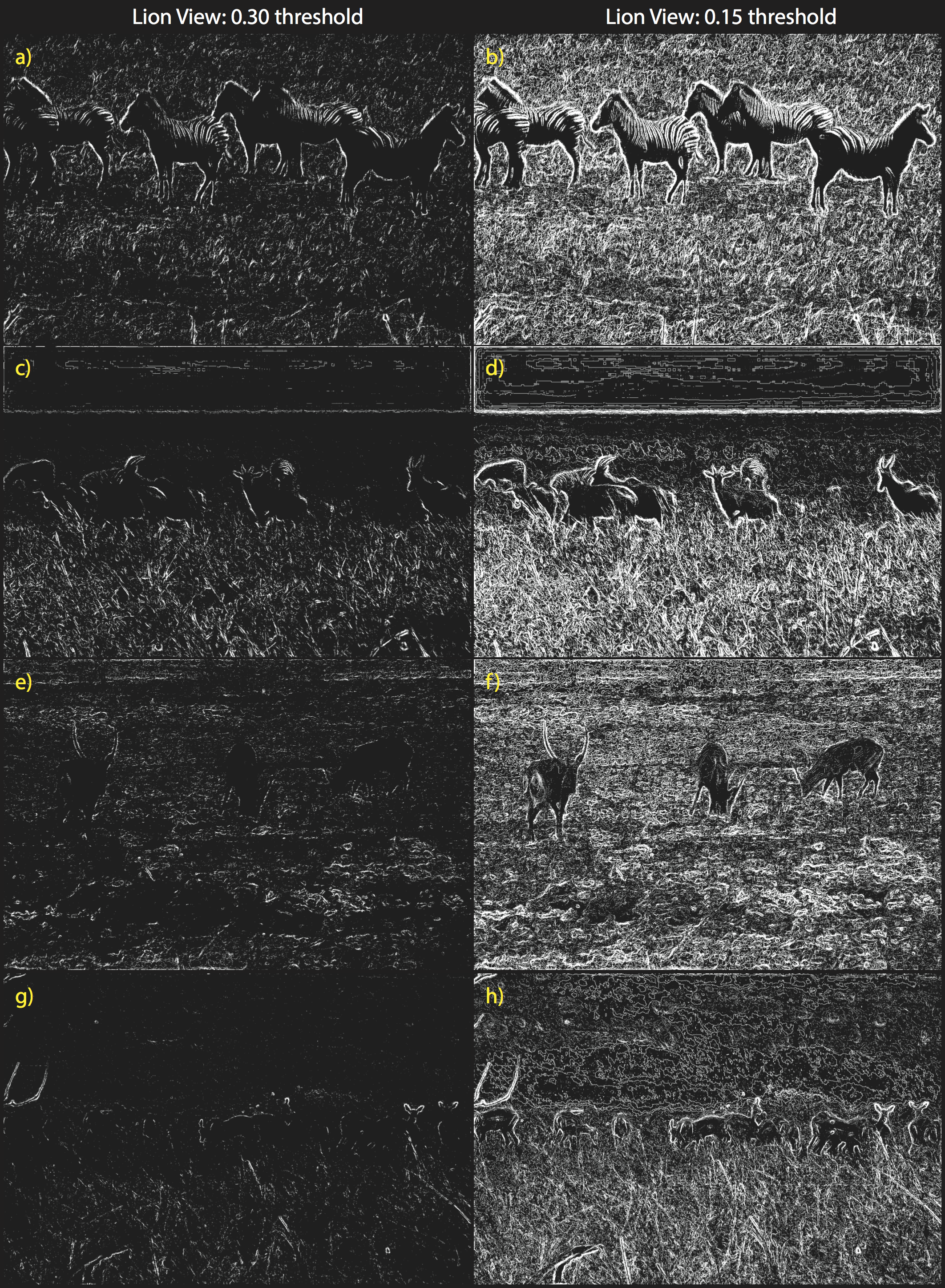

Supplement: S2 Fig — Images are modeled under a 0.30 threshold (left panels) and 0.15 threshold (right panels) for a lion’s visual system. (TIFF) [file pone.0145679.s004.tiff]

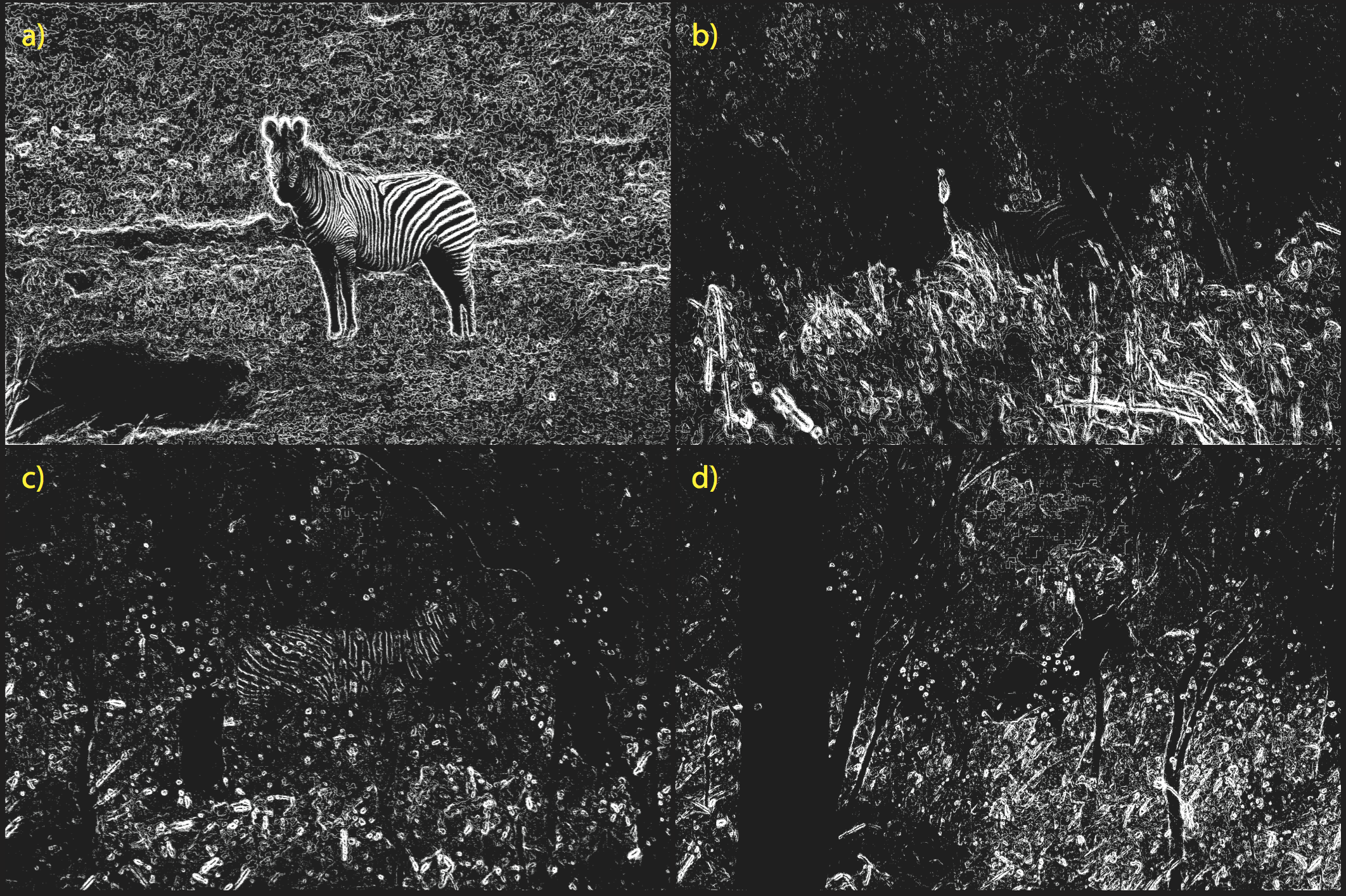

Supplement: S3 Fig — Images are modeled for a lion visual system under photopic conditions. (TIFF) [file pone.0145679.s005.tiff]
