# Supplementary material for: Zebra Stripes through the Eyes of Their Predators, Zebras, and Humans
Source: PLoS One. 2016 Jan 22;11(1):e0145679. doi: 10.1371/journal.pone.0145679 (PMC4723339; doi:10.1371/journal.pone.0145679)
Supplement: S1 Table — Values represent how much closer to the target the species listed vertically would need to be to resolve the same level of spatial detail as the species listed horizontally. (DOCX) [file pone.0145679.s006.docx]

**Supplemental Table 1.** Relative visual performance of different species under decreasing ambient light. Values represent how much closer to the target the species listed vertically would need to be to resolve the same level of spatial detail as the species listed horizontally.

|  |  | Human best (20/10 Snellen) | Zebra | Lion |
| --- | --- | --- | --- | --- |
| Daylight | Human 20/10 | 1 |  |  |
|  | Zebra | 2.58 | 1 |  |
|  | Lion | 4.47 | 1.74 | 1 |
|  | Hyaena | 7.48 | 2.91 | 1.67 |
| Dusk | Human 20/10 | 1 |  |  |
|  | Zebra | 1.86 | 1 |  |
|  | Lion | 3.02 | 1.63 | 1 |
|  | Hyaena | 5.07 | 2.71 | 1.67 |
| Moonless night | Human 20/10 | 1 |  |  |
|  | Zebra | 0.80 | 1 |  |
|  | Lion | 0.68 | 0.85 | 1 |
|  | Hyaena | 1.10 | 1.38 | 1.38 |
